# Focal accumulation of preribosomes outside the nucleolus during metaphase–anaphase in budding yeast

**DOI:** 10.1261/rna.061259.117

**Published:** 2017-09

**Authors:** Giulia Moriggi, Sonia G. Gaspar, Blanca Nieto, Xosé R. Bustelo, Mercedes Dosil

**Affiliations:** 1 Centro de Investigación del Cáncer and Instituto de Biología Molecular y Celular del Cáncer, CSIC-University of Salamanca, 37007 Salamanca, Spain; 2 Centro de Investigación Biomédica en Red de Cáncer (CIBERONC), Centro de Investigación del Cáncer, 37007 Salamanca, Spain; 3 Departamento de Bioquímica y Biología Molecular, University of Salamanca, 37007 Salamanca, Spain

**Keywords:** nucleolar functions, preribosomes, ribosome synthesis

## Abstract

*Saccharomyces cerevisiae* contains one nucleolus that remains intact in the mother-cell side of the nucleus throughout most of mitosis. Based on this, it is assumed that the bulk of ribosome production during cell division occurs in the mother cell. Here, we show that the ribosome synthesis machinery localizes not only in the nucleolus but also at a center that is present in the bud side of the nucleus after the initiation of mitosis. This center can be visualized by live microscopy as a punctate body located in close proximity to the nuclear envelope and opposite to the nucleolus. It contains ribosomal DNA (rDNA) and precursors of both 40S and 60S ribosomal subunits. Proteins that actively participate in ribosome synthesis, but not functionally defective variants, accumulate in that site. The formation of this body occurs in the metaphase-to-anaphase transition when discrete regions of rDNA occasionally exit the nucleolus and move into the bud. Collectively, our data unveil the existence of a previously unknown mechanism for preribosome accumulation at the nuclear periphery in budding yeast. We propose that this might be a strategy to expedite the delivery of ribosomes to the growing bud.

## INTRODUCTION

The synthesis of ribosomes starts with the assembly of the precursors of the 40S and 60S subunits in the nucleolus and, subsequently, progress along independent pathways that take place both in the nucleoplasm and the cytoplasm (for a scheme, see Fig. 2A). In the case of *Saccharomyces cerevisiae*, the initial assembly steps occur in a single nucleolus that is formed around the rDNA, a long locus present in chromosome XII that harbors a tandem array of approximately 150 polycistronic rRNA gene repeats (each containing a 5S rRNA and a 35S pre-rRNA sequence) (Nomura 2001). The first step of ribosome synthesis is the transcription by RNA polymerase I (Pol I) of the 35S pre-rRNA, the initial RNA precursor that contains the sequences for the mature 18S, 5.8S, and 25S rRNAs (Woolford and Baserga 2013). The 35S pre-rRNA nucleates the formation of a large 90S particle (also referred to as 90S preribosome or small subunit processome) composed of the U3 small nucleolar ribonucleoprotein (U3 snoRNP) and approximately 70 *trans*-acting factors that bind to the nascent transcript in a stepwise manner (Dragon et al. 2002; Grandi et al. 2002; Gallagher et al. 2004; Pérez-Fernández et al. 2007, 2011; Phipps et al. 2011; Chaker-Margot et al. 2015; Hunziker et al. 2016; Kornprobst et al. 2016; Zhang et al. 2016). Within the 90S particle, the 35S pre-rRNA is cleaved in a spacer region located between the 18S and the 5.8S rRNAs to yield a pre-40S particle and a pre-60S particle that will follow separate maturation routes and render the 40S and 60S ribosomal subunits, respectively (Henras et al. 2008; Kressler et al. 2010; Thomson et al. 2013; Woolford and Baserga 2013; Fernandez-Pevida et al. 2014; Henras et al. 2015; Nerurkar et al. 2015). The pre-40S particle undergoes few compositional changes while traveling through the nucleoplasm and, as a consequence, is rapidly exported to the cytoplasm (Schäfer et al. 2003, 2006). In contrast, the pre-60S particle has to go through extensive maturation steps in the nucleoplasm that involve the engagement of more than 40 *trans*-acting factors (Woolford and Baserga 2013; Nerurkar et al. 2015; Greber 2016; Wu et al. 2016). Once in the cytosol, both the pre-40S and pre-60S particles have to undergo final structural changes and quality control mechanisms before entering the pool of functional ribosomes (Strunk et al. 2011; Lebaron et al. 2012; Karbstein 2013; García-Gómez et al. 2014; Hector et al. 2014; Turowski et al. 2014).

A distinctive feature of *S. cerevisiae* is the exclusion of the rDNA from the rest of the genomic DNA and its confinement to a region close to the nuclear envelope opposite to the spindle pole body (Taddei and Gasser 2012). This localization depends, at least in part, on the tethering of the rDNA to the inner nuclear membrane through a network of proteins that include the cohibin complex (Csm1 and Lrs4), the CLIP complex (Heh1 and Nur1), and Sir2 (Mekhail et al. 2008). It is believed that this spatial separation ensures the stability of the highly repetitive rDNA sequences by restricting the accessibility of recombination factors (Mekhail et al. 2008; Taddei and Gasser 2012). It also facilitates the rapid formation of ribosomes due to the concentration of the ribosome manufacturing machinery within a well-defined nuclear subregion, the nucleolus. The formation of this organelle is a self-driven process initiated by the production of the rDNA-encoded 35S pre-rRNA precursor that, in turn, promotes the cotranscriptional recruitment of a large number of both ribosomal components and *trans*-acting factors (Oakes et al. 1993; Trumtel et al. 2000; Albert et al. 2011). In budding yeast, the concentration of the rDNA at one pole of the nucleus gives rise to one crescent-shaped nucleolus that abuts the nuclear envelope and occupies up to one-third of the total nuclear volume. Interestingly, the position of the nucleolus influences the spatial organization of specific genomic regions present in other chromosomes within the nucleus. For example, several tRNA-encoding genes that are scattered throughout the genome in different chromosomes cluster at the periphery of the nucleolus. It is believed that this process facilitates the coregulation of the ribosome and tRNA biosynthetic pathways (Thompson et al. 2003; Haeusler and Engelke 2006).

The dynamics of the nucleolus during the cell cycle is relatively well known. Chromosome XII becomes hypercondensed in mid-anaphase via the Cdc14 phosphatase-dependent down-regulation of Pol I that, in turn, allows the recruitment of condensin to the rDNA (Lavoie et al. 2004; Machín et al. 2005; Clemente-Blanco et al. 2009; Iacovella et al. 2015). Because the inhibition of the polymerase is transient, this condensation step does not have a major impact on the overall organization and integrity of the nucleolus. As a result, the nucleolar proteins remain in close proximity to the rDNA throughout the whole cell cycle. It is in late anaphase, upon segregation of the rDNA, that the nucleolus splits into two nucleoli that become symmetrically positioned in the mother and daughter cell nuclei (Bystricky et al. 2005). This late segregation step implies that ribosome production during most of the cell cycle originates in the mother cell. Consequently, the newly formed ribosomes carried over by the daughter cell must come from either the mother-cell cytoplasm or from preribosomes that are transported inside the nucleus from the mother-cell side to the bud-cell side. Despite the general acceptance of this model, however, little information is as yet available regarding the rates of preribosome export in different regions of the nuclear envelope and the mode of partition of ribosomes between mother and daughter cells. The potential presence of mechanisms to accelerate the delivery of ribosomes to the growing bud also remains unexplored. Interestingly, tRNA production is subject to spatial regulation during the cell cycle because tRNA genes become tethered to nuclear pore complexes (NPCs) in early mitosis through a Los1 exportin-mediated process (Chen and Gartenberg 2014). This mechanism is thought to expedite the export and accumulation of tRNAs in the cytoplasm. The reason for the loss of the spatial proximity between ribosome and tRNA production in mitosis is unknown.

In this work, we provide evidence indicating that yeast cells accumulate preribosomes at an extranucleolar center during metaphase–anaphase. The analyses of the subcellular localization, mode of formation, and composition of this extranucleolar center are consistent with a mechanism that, similarly to what happens with tRNA production, ensures the rapid delivery of ribosomes to the growing bud before effective nucleoli segregation.

## RESULTS

### Preribosomal components accumulate at a discrete body outside the nucleolus during the metaphase–anaphase transition

**FIGURE 1.**
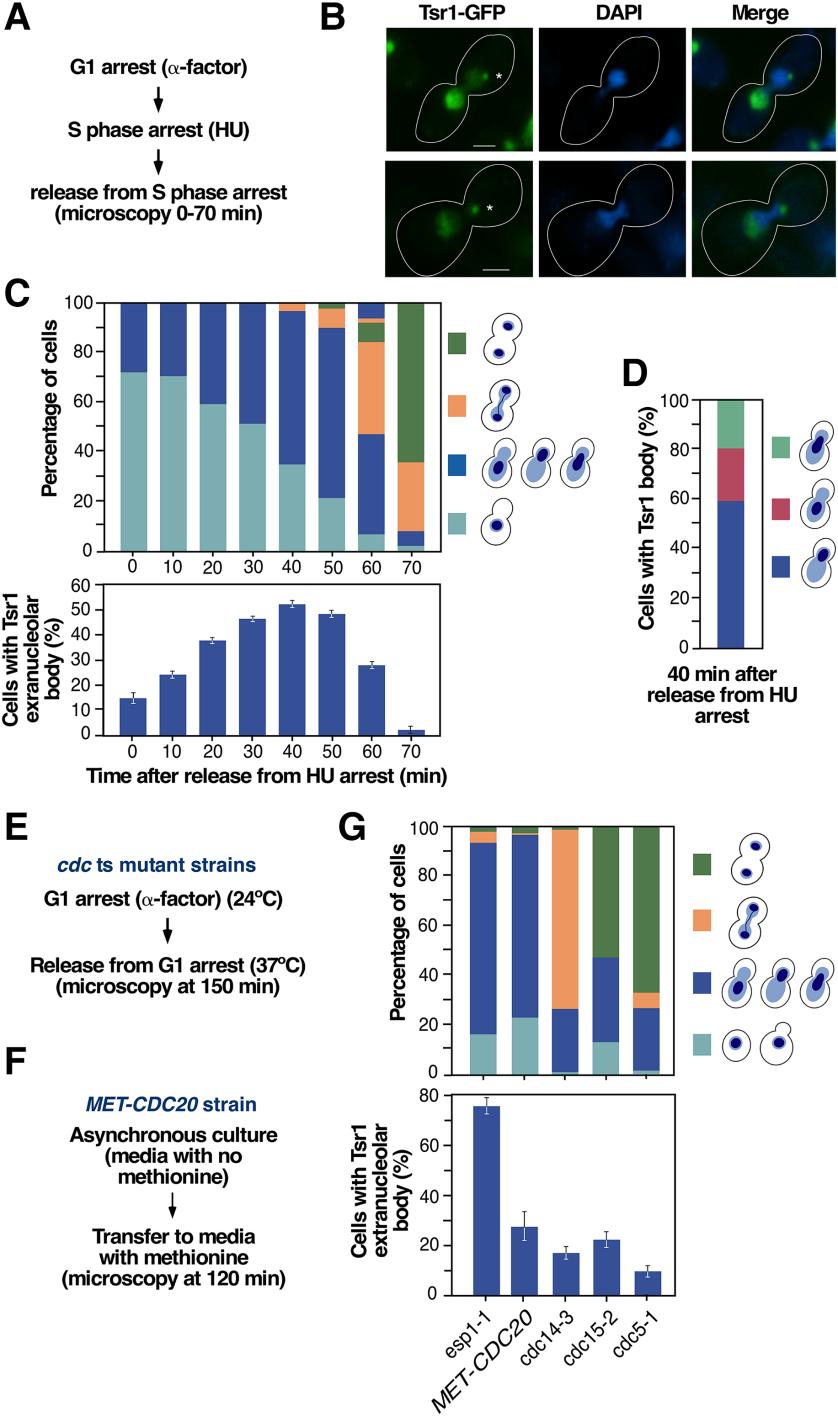
Tsr1-GFP localizes at an extranucleolar body in early mitosis. (*A*) Outline of the experimental plan to analyze the subcellular distribution of GFP-tagged Tsr1 in early mitosis. HU, hydroxyurea. (*B*) Representative images of Tsr1-GFP (green color) in cells transiting early mitosis. As in the rest of the figures, the time taken for imaging cells was 40 min upon the release of cells from hydroxyurea arrest. Scale bars, 2 µm. Asterisks indicate the position of the Tsr1^+^ extranucleolar body. (*C*) Quantification of cells at different cell-cycle stages using as criteria the position of the DNA mass (*top* panel) and of cells with Tsr1-GFP at the extranucleolar body upon release from hydroxyurea arrest (*bottom* panel). The types of cell morphology and position of the DNA mass are depicted on the *right*. (*D*) Quantification of differences in the positioning of the DNA mass in cells that exhibit the Tsr1^+^ extranucleolar body. The positioning types of the DNA mass are depicted on the *right*. (*E*,*F*) Outlines of the experimental plans used to analyze the presence of the Tsr1^+^ extranucleolar body in cells blocked at different stages of mitosis. ts, temperature-sensitive. (*G*) Quantification of cell-cycle stages using as criteria the positioning of the DNA mass (*top* panel) and of cells with Tsr1-GFP at the extranucleolar body (*bottom* panel) in the indicated cell-cycle-arrested strains. The types of cell morphology and positioning of the DNA mass are depicted on the *right*. Note that the *esp1-1* strain was analyzed when most cells are stalled at anaphase onset (*esp1-1* cells undergo aberrant mitosis when incubated for a long time at 37°C).

**FIGURE 2.**
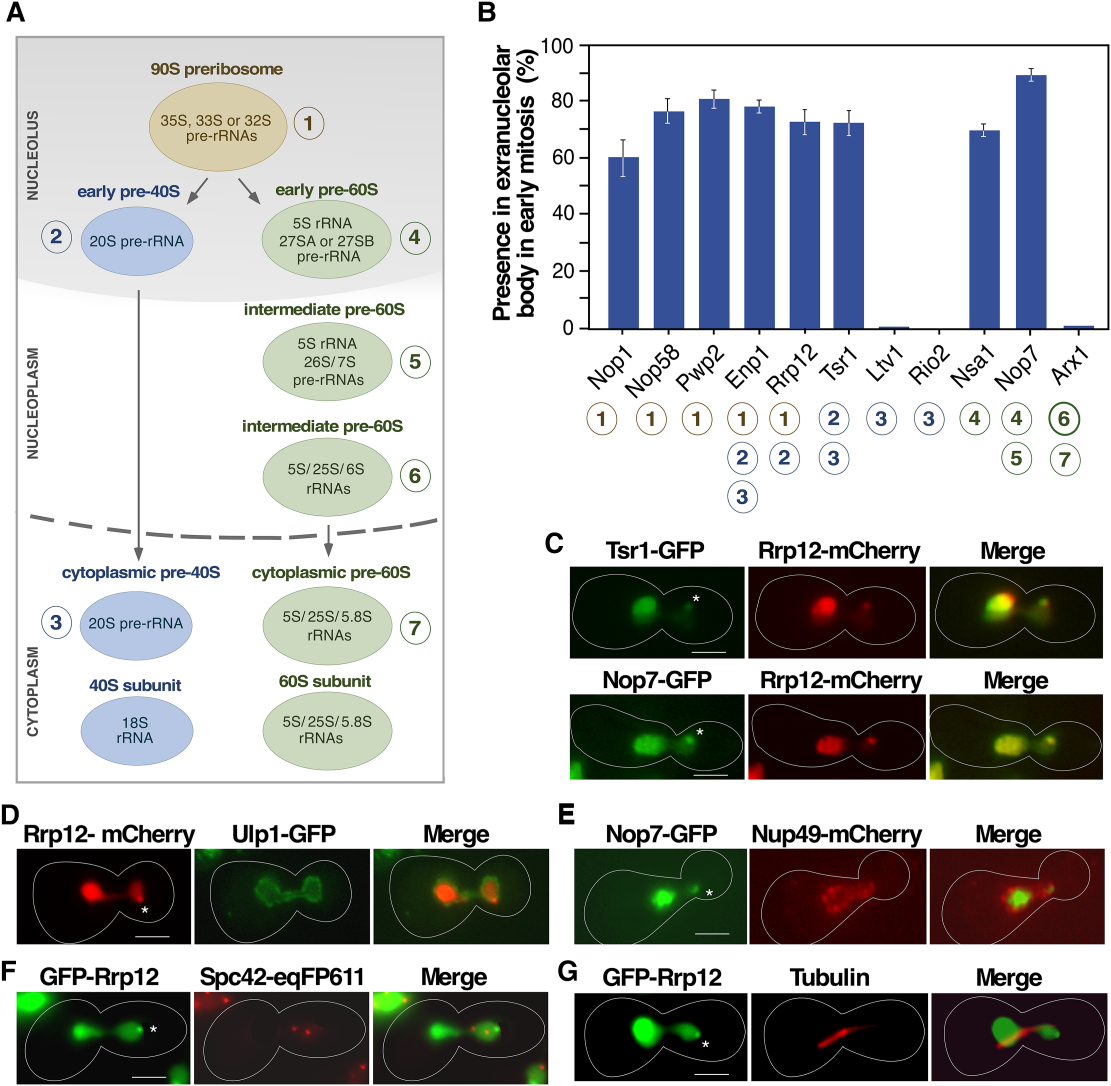
The Tsr1^+^ extranucleolar body contains both 40S and 60S preribosome particle components. (*A*) Scheme of the maturation of ribosomal subunits in *S. cerevisiae*. (*B*) Quantification of the presence in the extranucleolar body of the indicated GFP-tagged proteins in early mitosis cells. Circled numbers (*bottom*) indicate the specific association of those proteins with the preribosomal particles depicted in panel *A*. (*C*–*F*) Representative images of indicated GFP- (green color), mCherry- (red color), and eqFP611- (red color) tagged proteins in cells transiting early mitosis. (*G*) Representative image of endogenous tubulin (red color) detected by standard immunofluorescence techniques in cells expressing GFP-Rrp12 (green color). In panels *B*–*G*, the experiments were performed with cells 40 min upon release from hydroxyurea arrest following the scheme outlined in Figure 1A. In panels *C*–*G*, areas of colocalization are shown in yellow. Asterisks indicate the position of the extranucleolar preribosome body. Scale bars, 2 µm.

**FIGURE 3.**
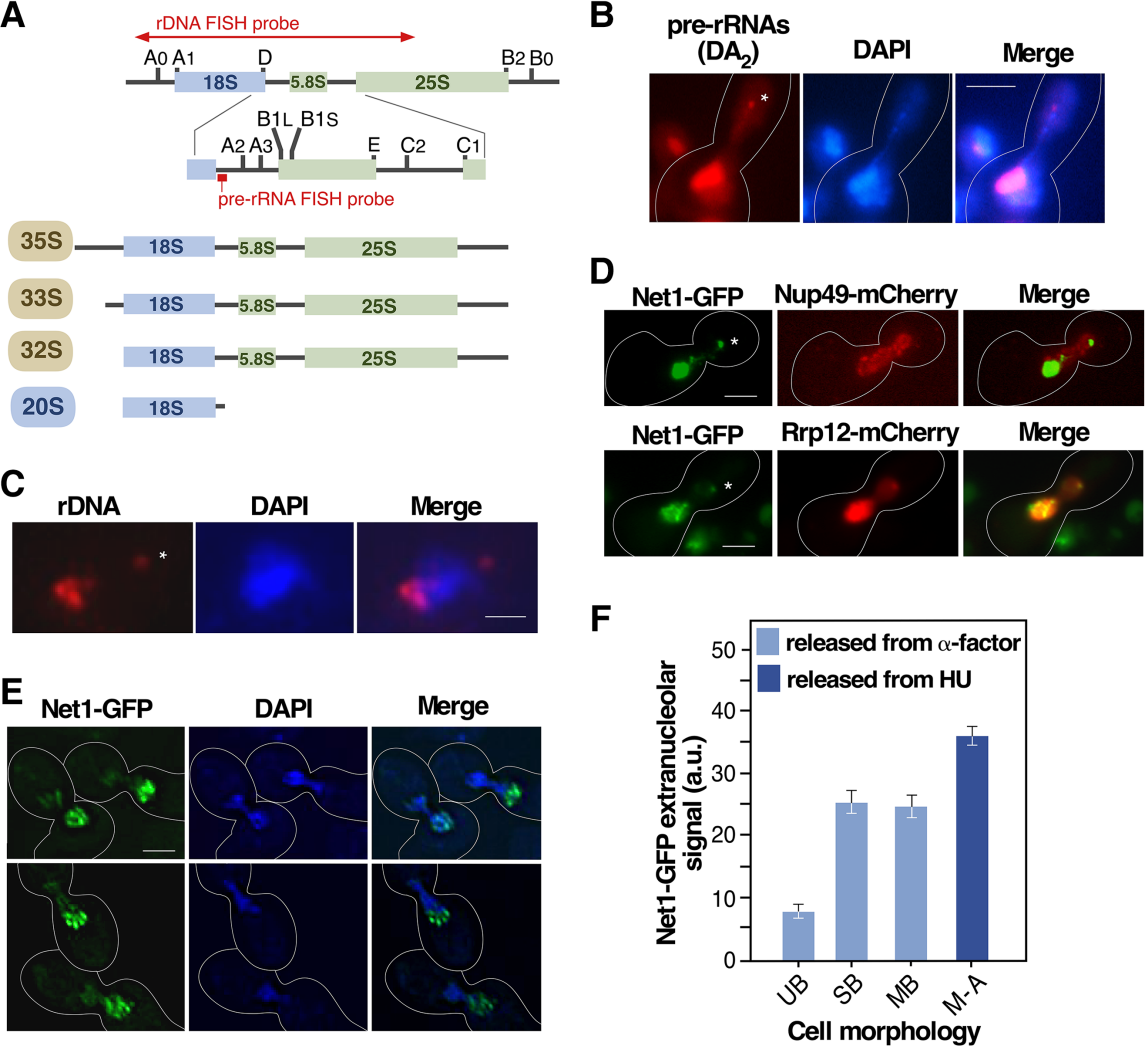
The extranucleolar preribosome body contains both pre-rRNA and rDNA. (*A*) Scheme of the structure of the 35S pre-rRNA and intermediate rRNA precursors detected in the RNA FISH analyses shown in panel *B*. The position of the ITS-1 pre-rRNA probe is indicated. The 35S, 33S, and 32S pre-rRNAs are present in 90S particles, and the 20S pre-rRNA is present both in nucleolar and cytoplasmic pre-40S particles (see preribosome maturation pathway in Fig. 2A). The region encompassed by the rDNA FISH probe used for the experiment shown in panel *C* is indicated. (*B*) Representative image of the extranucleolar localization (indicated by asterisk) of pre-rRNAs in *esp1-1* cells arrested in metaphase–anaphase that were analyzed by RNA FISH using a probe for the ITS-1 region. (*C*) Representative image of rDNA localization in *esp1-1* cells arrested in metaphase–anaphase that were analyzed by DNA FISH. (*D*) Representative images of the subcellular localization of indicated GFP- (green color) and mCherry (red color)-tagged pairs of proteins in cells transiting metaphase–anaphase 40 min upon release from hydroxyurea arrest. (*E*) Representative images of *net1-GFP*-expressing cells transiting early mitosis upon release from α-factor arrest (*top* panels), and of *esp1-1/net1-GFP* cells arrested in metaphase–anaphase (*bottom* panels) taken under slow-bleach low-resolution conditions. (*F*) Quantification of Net1-GFP extranucleolar fluorescence in unbudded (UB), small bud (SB, bud size < 0.3× mother cell diameter), and medium bud (MB, bud size 0.3–0.6× mother cell diameter) wild-type cells released from α-factor arrest and wild-type cells at metaphase–anaphase (M–A) upon release from hydroxyurea arrest. HU, hydroxyurea. Scale bars (*A*–*C*), 2 µm.

Tsr1 is a ribosome biogenesis factor essential for 40S subunit formation. Recent studies indicate that this protein works as a molecular gatekeeper that binds to pre-40S particles in the nucleolus to mask structural sites that have to become accessible only during final maturation in the cytoplasm (Strunk et al. 2011; McCaughan et al. 2016). Consistent with earlier studies (Schäfer et al. 2003; Moriggi et al. 2014), we found using epifluorescence microscopy analyses that a version of Tsr1 tagged at its carboxyl terminus with green fluorescent protein (GFP) localizes in the nucleolus when monitored both in asynchronously growing cells (data not shown) and in cells going through mitosis after synchronization in S phase with hydroxyurea (Fig. 1A,B). However, we unexpectedly found the consistent accumulation during early mitosis of Tsr1-GFP in a well-defined extranucleolar site (referred to hereafter as “extranucleolar body”) that localizes in the bud side of the nucleus in a position opposite to the nucleolus (Fig. 1B). Further analyses using hydroxyurea-arrested cells indicated that the accumulation of Tsr1 in the extranucleolar body peaks ≈40 min upon the release of cells from the arrest, a time that coincides with the progression of cells from metaphase to early anaphase (Fig. 1C). Consistent with this, all cells displaying the Tsr1^+^ extranucleolar body show typical features of the metaphase–anaphase transition, including an elongated nucleus extending from the mother to the bud, the localization of the bulk of DNA near the bud neck, a short (2–3.5-µm-long) mitotic spindle, and an early anaphase-like rDNA morphology (Fig. 1B,D; pictures of the mitotic spindle and rDNA morphology are shown below in Figs. 2 and 3, respectively). Similar findings, but with weaker fluorescent signals, were made using cells released from α-factor-induced G_1_ arrest (data not shown), indicating that the localization of Tsr1-GFP in this extranucleolar body is not an experimental artifact derived from the hydroxyurea synchronization protocol (see Materials and Methods). Confirming its association with the metaphase–anaphase transition, we found that the Tsr1^+^ extranucleolar body becomes highly enriched in mutant (*esp1-1*) cells stalled at the anaphase onset (Fig. 1E,G). In contrast, this increase does not occur in other mutant strains blocked at earlier or later stages of mitosis, such as metaphase (*MET-CDC20*), anaphase–telophase (*cdc14-3*), or telophase (*cdc15-2* and *cdc5-1*) (Fig. 1E–G).

The foregoing results suggested the possibility of a hitherto unknown location for ribosome synthesis factors during early mitosis. To investigate this possibility, we first monitored the subcellular localization of a collection of *trans*-acting factors belonging to the 40S or 60S ribosomal subunit synthesis pathways (Fig. 2A; Henras et al. 2008; Woolford and Baserga 2013). Those included 90S preribosome-specific components (Nop1, Nop58, and Pwp2), 90S preribosome proteins that remain in the pre-40S particle until the final maturation steps in the cytoplasm (Enp1), a 90S and early pre-40S particle component (Rrp12), and cytoplasmic pre-40S particle elements (Ltv1, Rio2). We also included integral components of early (Nsa1), early-intermediate (Nop7), and intermediate-cytoplasmic (Arx1) pre-60S particles. To facilitate our studies, these proteins were expressed as GFP-tagged versions from their respective genetically modified endogenous locus. As a control to verify that the GFP tag itself does not influence protein localization, all data obtained from live-cell microscopy analyses in cells expressing Nop7-GFP were confirmed with anti-MYC immunofluorescence analyses in cells expressing Nop7-MYC (data not shown). All preribosomal factors interrogated exhibited the expected subcellular localization in the nucleolus (Nop1, Nop58, Pwp2, Nsa1), nucleolus plus nucleoplasm (Enp1, Rrp12, Tsr1, Nop7), and nucleoplasm plus cytoplasm (Ltv1, Rio2, Arx1) both in nonsynchronized and synchronized cells (data not shown). However, similarly to the data obtained with Tsr1-GFP, we found that all the 90S preribosome-specific (Nop1, Nop58, Pwp2), early-intermediate pre-40S (Enp1, Rrp12), and early-intermediate pre-60S (Nsa1, Nop7) components display a transient accumulation during metaphase–anaphase in an extranucleolar body-like structure (Fig. 2B,C). This subcellular localization is not observed in the case of late pre-40S and pre-60S maturation factors such as Ltv1, Rio2, and Arx1 (Fig. 2B). Additional subcellular colocalization analyses using cells expressing specific pairs of GFP- and mCherry-tagged proteins confirmed that 90S preribosome (Rrp12), pre-40S (Rrp12, Tsr1), and pre-60S (Nop7) factors are present at the Tsr1^+^ extranucleolar body (Fig. 2C).

The position of this extranucleolar body relative to the 4′,6-diamidino-2-phenylindole (also known as DAPI)-stained DNA suggested a close proximity to the nuclear envelope (see above, Fig. 1B). Consistent with this, we found that an Rrp12-mCherry protein present in that body localizes proximally to both associated (Ulp1-GFP) and intrinsic (Nup49) NPC markers (Fig. 2D,E). The punctate nature of the extranucleolar body also suggested that it might be the spindle pole body. However, we ruled out this possibility because Rrp12-mCherry does not colocalize with a spindle pole body component (Spc42-eqFP611) (Fig. 2F) or tubulin (Fig. 2G). Consistent with this, we could not find any correlation between the positioning of the extranucleolar body and the orientation of the mitotic spindle or the movement of the spindle pole body (i.e., see Fig. 2G). These results indicate that there is a focal accumulation of both early and intermediate preribosomal proteins outside the nucleolus in a region of the nuclear envelope at the bud during early mitosis.

### The extranucleolar body contains both pre-rRNAs and rDNA

To determine whether the proteins located at the extranucleolar body form part of maturing preribosomal complexes, we examined if pre-rRNA species were present in that site using fluorescent in situ hybridization analyses (FISH). To this end, we utilized a Cy3-labeled probe specific for the pre-rRNA D-A_2_ segment (Fig. 3A). This region maps within the 35S, 33S, 32S, and 20S pre-rRNAs and, therefore, can be used to decorate the 90S and pre-40S preribosomes (see above, Fig. 2A). Consistent with this, we found that this probe can label the nucleoli of all cells examined (Fig. 3B). However, we also found that it decorates an extranucleolar spot similar to those seen with GFP-tagged preribosomal proteins (Fig. 3B). These results indicate that this body harbors functional preribosomal particles and, therefore, that it might represent an active site of ribosome synthesis. If this hypothesis were correct, the body should contain rDNA. Using FISH analyses, we observed a weak rDNA signal at a site separated from the bulk of the rDNA that resembled the extranucleolar body (Fig. 3C). To better define the localization of the rDNA and reveal its colocalization with protein markers, we performed microscopy analyses of a GFP-tagged version of Net1. This protein is known to be tightly associated with the rDNA throughout the cell cycle and has been fully validated as an in vivo rDNA marker (Machín et al. 2005). As seen in Figure 3D, the rDNA exhibits the expected “puff-like” morphology and mother-cell localization of cells transiting metaphase–anaphase (Fuchs and Loidl 2004; Machín et al. 2005). In addition, a focal concentration of rDNA inside the bud is observed, at a spot on the nuclear envelope (decorated by Nup49-mCherry) that colocalizes with the preribosome extranucleolar body (visualized by the Rrp12-mCherry marker). As in the other experiments of this work, this localization was only observed in metaphase–anaphase. Interestingly, we observed that the rDNA present at the extranucleolar body is often located at the distal end of a thread-like structure that originates in the bulk of rDNA of the mother cell. These threads can be visualized using both 4′,6-diamidino-2-phenylindole staining (Fig. 3B) and Net1-GFP epifluorescence (Fig. 3D, upper panel), thus suggesting that a discrete region of the rDNA moves away from the nucleolus of the mother cell in early mitosis to form the extranucleolar body. In agreement with this interpretation, quantitation of the percentage of extranucleolar Net1-GFP indicated that discrete regions of the rDNA start moving outside the nucleolus during the initial stages of cell division (Fig. 3E) and, to a much larger extent, during the metaphase–anaphase transition (Fig. 3F). These results suggest that the increased mobility of rDNA during early mitosis might contribute to the formation of a small extranucleolar preribosome production center in the bud.

### Preribosome extranucleolar body formation follows nonredundant mechanisms to those used for the subcellular localization of nucleolar rDNA and tDNAs

**FIGURE 4.**
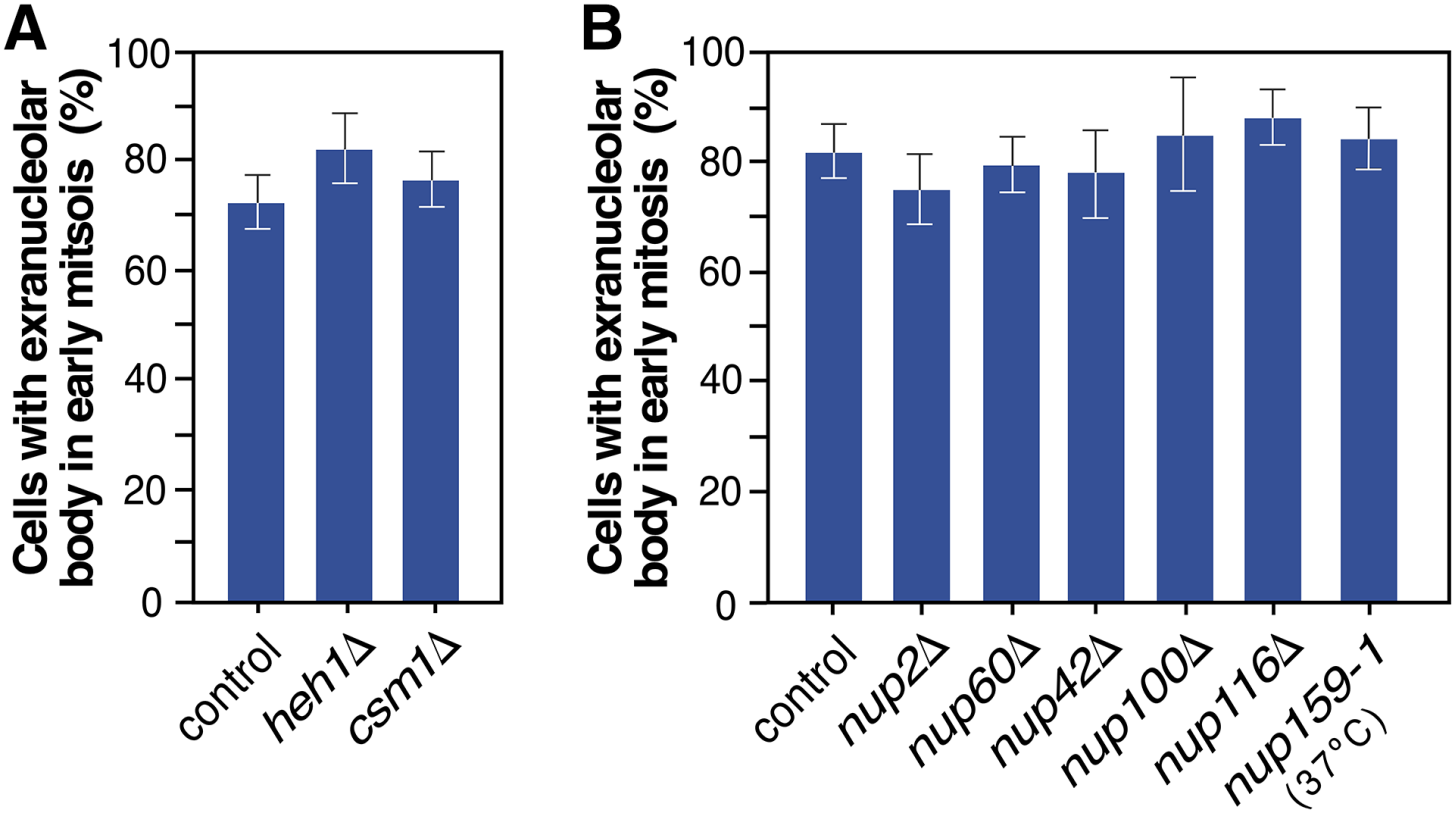
The formation of the extranucleolar body does not involve the same mechanisms used for tethering the rDNA and tDNA to the nuclear envelope. (*A*,*B*) Quantification of the presence of Nop7-GFP at the extranucleolar body in early mitosis cells from the indicated yeast strains. The experimental plan was the same as the one outlined in Figure 1A for wild-type cells, except in the case of the *nup159-1* cells, which were arrested in G_1_ with α-factor at 24°C and then shifted to 37°C upon release from the G_1_ arrest.

To assess whether the localization of the rDNA in the bud followed mechanisms similar to those used for the perinuclear positioning of the bulk of rDNA, we tested the effect of disrupting the *HEH1* and *CSM1* genes in the formation of the extranucleolar body. These genes encode components of the CLIP and cohibin complexes that are essential for the normal tethering of the nucleolus-localized rDNA to the nuclear envelope (Mekhail et al. 2008). We found no alterations in the positioning of the nucleolus at the mother side upon deletion of any of those two genes (data not shown). The timing, formation, morphology, and placing of the extranucleolar body are also normal (Fig. 4A and data not shown). Likewise, we did not detect any change in the formation of the extranucleolar body upon the loss of either Nup2 or Nup60 (Fig. 4B), two nucleoporins known to be involved in the correct tethering of tRNA genes to NPCs in early mitosis (Chen and Gartenberg 2014). Similar data were obtained in cells deficient in nucleoporins (Nup42, Nup100, Nup116, and Nup159) that directly interact with Crm1 (Fig. 4B), an exportin that plays a key role in the nuclear export of preribosomal particles (Neville et al. 1997; Hodge et al. 1999; Oeffinger et al. 2004; Yu et al. 2008; Light et al. 2010). Taken together, these results indicate that the mechanisms underlying the formation of the extranucleolar preribosome body are distinct from those involved in the positioning of the nucleolar rDNA in the mother cell and the localization of the tDNA at the nuclear envelope in early mitosis.

### The Crm1 exportin is required for the accumulation of preribosomes at the extranucleolar body

**FIGURE 5.**
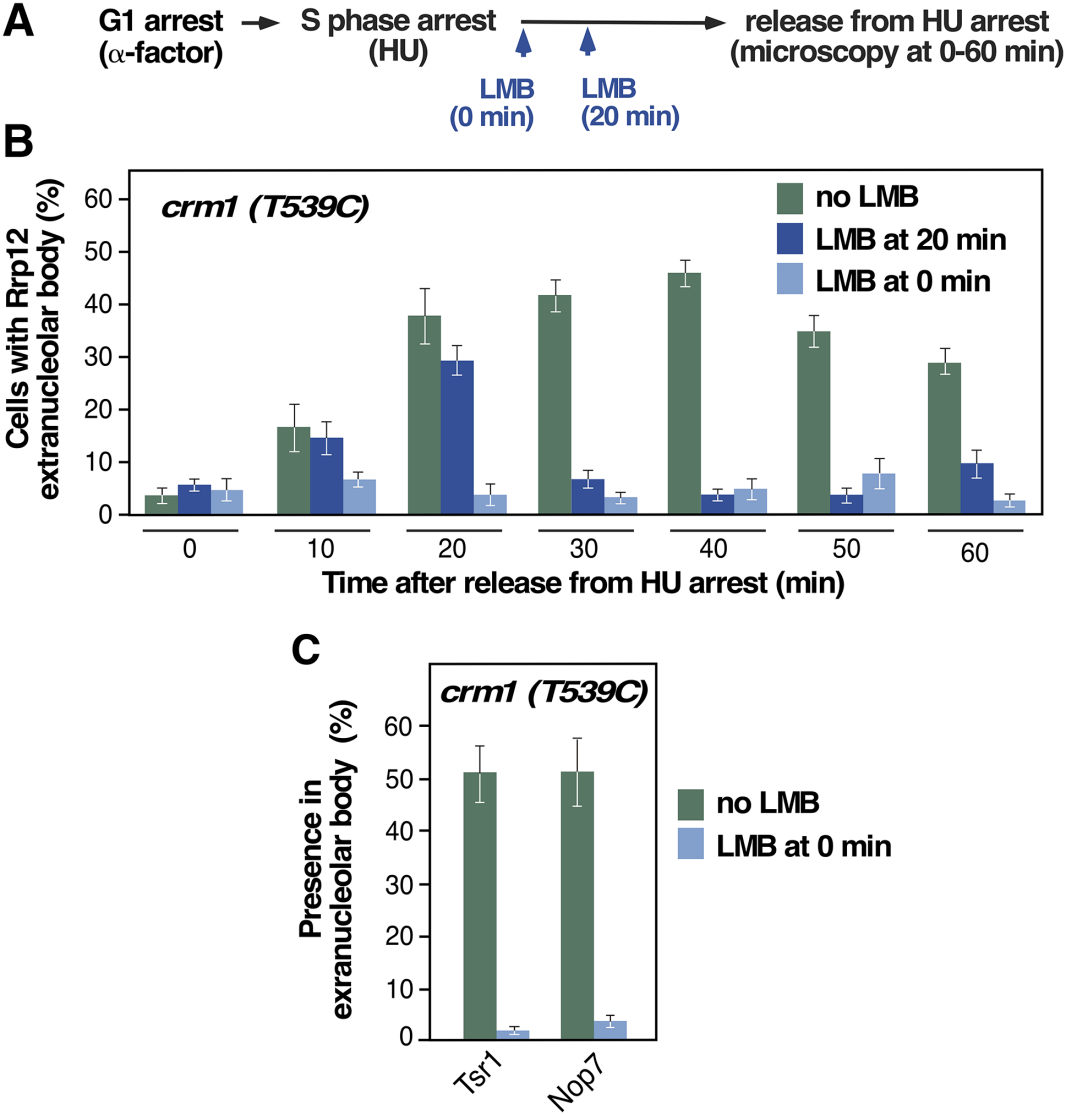
The extranucleolar preribosome body is enriched in maturing complexes primed for nuclear export. (*A*) Scheme of the experimental plan used to analyze the formation and maintenance of the extranucleolar preribosome body upon leptomycin B (LMB)-mediated Crm1 inactivation in *crm1-T539C* cells. HU, hydroxyurea. (*B*) Quantification of cells with GFP-Rrp12 localized at the extranucleolar body upon release from HU arrest under the indicated experimental conditions (*inset*). (*C*) Quantification of the percentage of GFP-Tsr1 and GFP-Nop7 localized at the extranucleolar body upon release from HU arrest for 40 min in the presence and absence of LMB.

The concurrent presence of preribosomal particles and rDNA in the extranucleolar body suggested that this could be an active site of ribosome synthesis. If that were the case, this body has to contain all the machinery required for the coordinated integration of the intranucleolar and nucleoplasmic steps involved in the synthesis of both ribosomal subunits. To explore this issue, we evaluated the role of the Crm1 exportin in the formation of the extranucleolar body. This exportin is thought to participate in the integration of preribosome assembly and nuclear export during 40S subunit synthesis by priming the emerging pre-40S particles in the nucleolus for rapid nuclear exit (Moriggi et al. 2014). To assess the role of Crm1 in extranucleolar body formation, we performed cell-cycle synchronization studies on cells harboring a leptomycin B-sensitive Crm1 mutant (T539C) (Fig. 5A). As a readout, we examined the subcellular localization of the pre-40S factor Rrp12 either upon the release of cells from the S-phase arrest or 20 min thereafter (Fig. 5A). With this strategy, we could assess the effect of Crm1 inhibition in the incorporation (0 time point upon arrest release) and maintenance (20 min postarrest release) of Rrp12 in the extranucleolar body, respectively. Using this approach, we found that these two steps were Crm1-dependent (Fig. 5B). Furthermore, similarly to what was found for Rrp12, the inhibition of Crm1 blocked the accumulation of two other preribosomal proteins, the pre-40S factor Tsr1 and the pre-60S factor Nop7, in the body (Fig. 5C). These results indicate that Crm1 function is required for the focal concentration of 40S and 60S preribosomes at the extranucleolar body during mitosis, further suggesting that it might be a site that contains preribosomes primed for nuclear export.

### Extranucleolar body localization can be used as a bona fide indicator for the active entry of *trans*-acting factors in the ribosome maturation pathway

**FIGURE 6.**
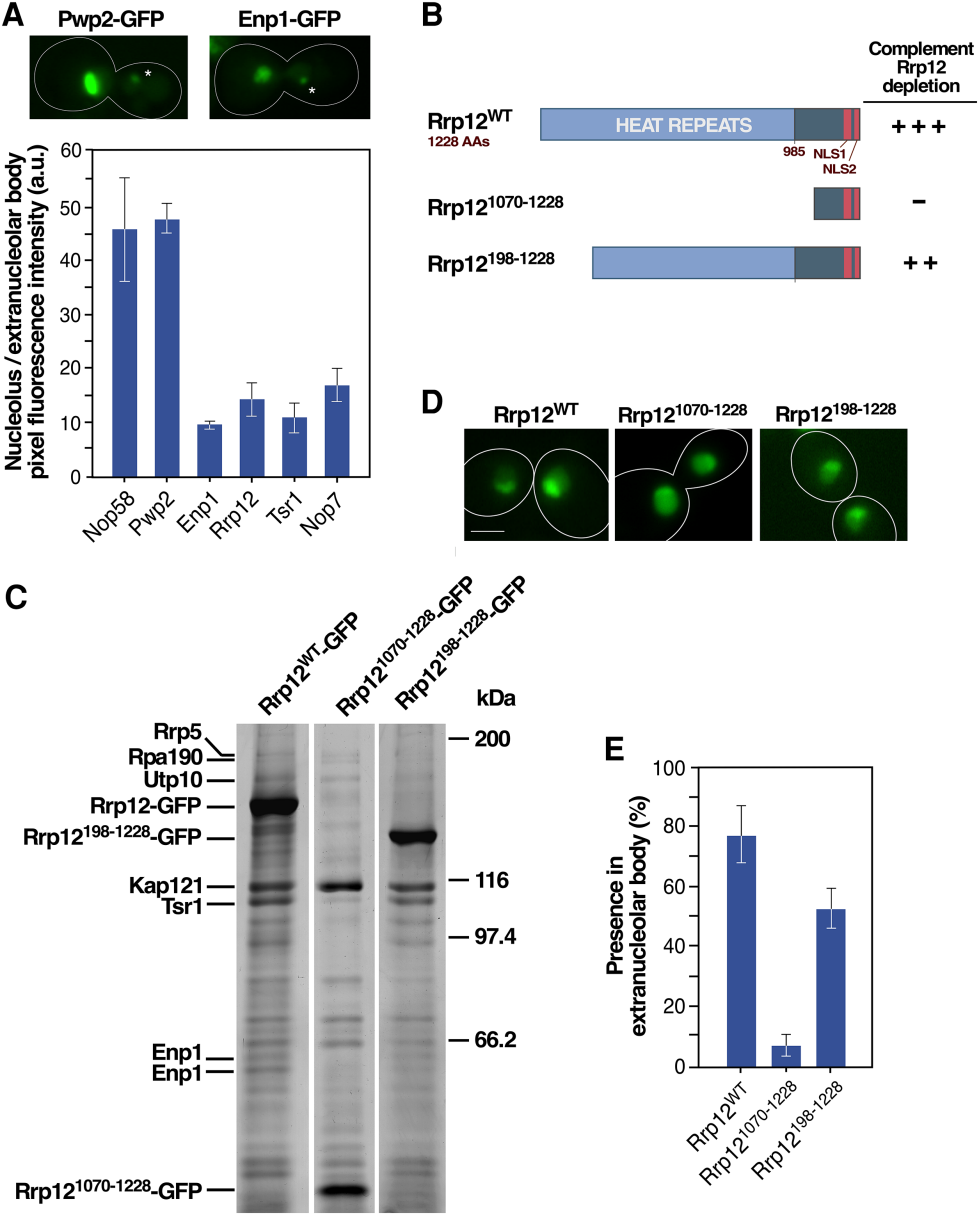
Protein localization at the extranucleolar body is a bona fide indicator of entry in the ribosome maturation pathway. (*A*) (*Top*) Representative images of the localization of indicated GFP-tagged proteins in the nucleolus and extranucleolar body (indicated by an asterisk). (*Bottom*) Quantification of the average fluorescence intensity per pixel detected for the indicated GFP-tagged proteins (*x*-axis) in the nucleolus and extranucleolar body. (*B*) Summary of the capacities of wild type and amino-terminal deleted versions of Rrp12 to complement the loss of Rrp12. +++, full complementation; ++, partial complementation; –, no complementation. (*C*) Electrophoretic analysis of proteins that copurify with GFP-tagged versions of Rrp12 (listed across the *top*). Proteins identified by mass spectrometry are shown on the *left*. Molecular weight markers are shown on the *right*. (*D*) Representative images of the localization of the indicated GFP-tagged versions of Rrp12 in asynchronously growing cells. Scale bar, 2 µm. (*E*) Quantification of the localization of the indicated Rrp12 versions at the extranucleolar body in cells transiting early mitosis 40 min upon release from hydroxyurea arrest. Scale bars (*A* and *D*), 2 µm.

We consistently noticed during the course of the foregoing analyses that the ratio of intra- versus extranucleolar epifluorescence signals derived from GFP-tagged proteins is always lower in 90S (i.e., Nop58 and Pwp2) (Fig. 2A) than in early-intermediate (Enp1, Rrp12, Tsr1, Nop7) (Fig. 2A) components (Fig. 6A). A possible explanation for these data is that the extranucleolar body has faster kinetics of production of pre-40S and pre-60S particles from 90S preribosomes than the nucleolus, thus leading to a relatively reduced concentration of 90S preribosomes in that site. We reasoned that this feature could be used as a tool to assess the active participation of *trans*-acting factors in ribosome biogenesis. To explore this idea, we decided to compare the localization of the wild-type and two amino-terminal deleted (Rrp12^1070-1228^, Rrp12^198-1228^) versions of the 90S/pre-40S component Rrp12 (Fig. 6B) in the nucleolus and in the extranucleolar body. Complementation analyses showed that Rrp12^WT^ and Rrp12^198-1228^ can fully and partially rescue the viability of Rrp12-depleted cells (Fig. 6B), respectively. In contrast, the short Rrp12^1070-2108^ fragment is nonfunctional (Fig. 6B). Consistent with these data, proteomic analyses indicate that Rrp12^WT^ and Rrp12^198-1228^, but not Rrp12^1070-1228^, can form stable interactions with pre-40S *trans*-acting factors such as Tsr1 and Enp1 (Fig. 6C). Despite these marked differences in functionality, we observed that the three Rrp12 versions are adequately localized in the nucleolus (Fig. 6D). This suggests that the nucleolar localization shown by Rrp12^1070-1228^ probably reflects an interaction with rDNA, preassembly subcomplexes or other nucleolar proteins outside preribosomal particles rather than an active involvement in the 40S subunit synthesis. In contrast, the localization in the extranucleolar body does seem to represent a good readout for protein functionality as evidenced by the detection of Rrp12^WT^ and Rrp12^198-1228^, but not Rrp12^1070-1228^, in that site in metaphase–anaphase (Fig. 6E). These results indicate that the extranucleolar body localization represents a more unequivocal biological parameter than nucleolar detection to assess the functionality of *trans*-acting factors in the ribosome synthesis pathway. They are also consistent with the notion that the extranucleolar body can be an active site of assembly and subsequent maturation of preribosomes.

## DISCUSSION

It has long been established that the nucleolus stays in the mother-cell side of the nucleus during most of the mitotic cycle in *S. cerevisiae*. As a result, it is assumed that the bulk of ribosomes inherited by the daughter cell have to be originated in the mother-cell side. In this work, we provide evidence showing that the spatial distribution of ribosome precursors during mitosis is more complex than previously thought in budding yeast. Thus, we have found an accumulation of preribosomes at a small extranucleolar site located in the bud side of the nucleus. This site is observed in the transition between metaphase and anaphase and can be detected by light microscopy as a small body placed at the nuclear envelope. Its small size, highly transient nature, and poor detection in 4′,6-diamidino-2-phenylindole-stained yeast cells might explain why this body has not been detected before. In fact, its discovery in this work was a serendipitous result derived from studies initially focused on the functional characterization of the early preribosome components Tsr1 and Rrp12 during the cell cycle. Although a demonstration of active pre-rRNA synthesis at the extranucleolar body has not been possible due to technical limitations, several lines of evidence strongly suggest that this is a center of active ribosome production: (i) It contains rDNA and pre-rRNA, two founding components of nucleolar-organizing regions that drive the recruitment of ribosome synthesis machinery. (ii) It harbors *trans*-acting factors present in preribosomal particles that eventually render mature 40S and 60S ribosomal subunits. (iii) It contains maturing preribosomes apparently primed for nuclear export, as assessed by its dependency on the activity of the Crm1 exportin. Our data also suggest that this site might be producing ribosomes with faster kinetics than the nucleolar-localized sites, as inferred from the higher ratio of accumulation of early-intermediate pre-40S and pre-60S (i.e., Enp1, Rrp12, Tsr1, Nop7) versus 90S (i.e., Nop58 and Pwp2) particle components.

What might be the reason for the accumulation of preribosomes at an extranucleolar site? A possible explanation is that, similarly to the case of tRNA production, budding yeast resort to the localized production of ribosomes at the nuclear envelope to facilitate their rapid export to the cytoplasm of the growing bud. Given the focal nature of the extranucleolar body, its role could be related to some specific functional needs of the growing bud that require a concentrated supply of ribosomes before the segregation of the nucleolus. Such needs might include, for example, the colonization by ribosomes of the reticulum endoplasmic tubules that are undergoing expansion and polarized growth from the perinuclear area to the bud cortex and/or the binding of ribosomes to mRNA complexes that are targeted for transport to the bud tip to be translated there (Ouellet and Barral 2012; Buxbaum et al. 2015).

The mode of formation and maintenance of this extranucleolar body is still unclear. Our results indicate that its formation might depend on the docking at the nuclear membrane of short segments of rDNA that are projected from the mother to the bud side of the dividing cell. These projections occur in early mitosis, a time in which the rDNA locus is known to be highly mobile and dynamic (see also Fig. 3C,D; Torres-Rosell et al. 2007; Miyazaki and Kobayashi 2011). Consistent with this idea, it is known that individual rDNA repeats occasionally exit the nucleolus in budding cells (Torres-Rosell et al. 2007). Another possibility is that extrachromosomal rDNA circles that occasionally pop out from chromosome XII could nucleate the extranucleolar body. However, we do not favor this possibility because published evidence indicates that such circles remain confined within the mother cell during mitosis (Sinclair and Guarente 1997). Regardless of the mechanism involved, one interesting feature is that these bodies always appear as a single bud-localized spot positioned opposite to the mother-cell nucleolus. The reason for the presence of a single body and for its unique location is as yet unclear. On the one hand, it is possible that these features are due to some regulated mechanism that ensures the polarized and stable anchoring of an rDNA segment in a specific subregion of the nuclear envelope present in the bud. On the other hand, it could be that the nucleation of this site is a purely stochastic process. For example, one possible scenario is that only a fraction of the many rDNA subregions moving out of the nucleolus are able to form a strong and stable contact with the nuclear envelope to eventually build the extranucleolar body. It is also possible that other extranucleolar bodies could pass unnoticed in our experiments due to a low accumulation of preribosomes that makes them undetectable by the techniques used in this study. We currently favor the “regulated mechanism” model, because it is the only one that can explain the consistent detection of a single spot in a defined location within the nuclear envelope in the extensive number of cells tested so far in our experiments. The mechanistic aspects involved in the actual docking of the rDNA at the nuclear envelope also remain unclear. Based on the precedent of the exportin-mediated association of tDNAs to nuclear pore complexes, one feasible mechanism is through the participation of an export factor. In this context, Crm1 is a good candidate as evidenced by the deleterious effect of its inhibition on the formation of the extranucleolar body. However, it is as yet unclear whether this effect is due to the implication of Crm1 in the anchoring of the rDNA to the nuclear envelope, the concentration of emerging preribosomes at the nuclear envelope, or both processes at the same time. Our attempts to further explore this issue have been hampered by the inability to visualize the rDNA at the extranucleolar body in the Crm1^T539C^ strain even in the absence of leptomycin B. The reason for this is unknown, although it must be noted that the Net1-GFP rDNA signal in the extranucleolar body is rather weak even when monitored in wild-type controls. Due to this, any minor reduction in fluorescence caused, for example, by slight decreases in the residence time of proteins will make the extranucleolar bodies undetectable with the techniques used in this study. In the case of the tDNAs, the model is that the exportin (Los1) promotes the contact of tDNAs with nucleoporins (Nup60 and Nup2) at nuclear pore complexes. In the case of the rDNA, although a possible interaction with nucleoporins remains an open possibility, our experiments have ruled out the specific involvement of known Crm1- and tDNA-binding nucleoporins in the docking of the extranucleolar body. Our experiments are also incompatible with the implication of the perinuclear rDNA tethering network in this process. Further genetic and biochemical experiments will be required to fully understand the mechanism of formation and regulation of this new site of ribosome production in yeast.

In addition to its biological implications, our work has shown that the localization in the extranucleolar body can be used as a good biological readout to assess the functionality of specific preribosomal factors in ribosome synthesis. In fact, this readout provides more faithful and clear-cut information than the detection of the same proteins in the nucleolus. Importantly, this method is experimentally very simple and can be easily implemented as a routine test to interrogate the activity of any desired preribosomal factor or mutant in ribosome biogenesis.

In conclusion, our data demonstrate the existence of a previously unknown mechanism for small-scale preribosome accumulation at the nuclear envelope in *S. cerevisiae*. We postulate that this strategy might be used for the efficient delivery of ribosomes to sites of rapid growth or to specific cellular regions of high mRNA translation activity. It is possible that extranucleolar sites of preribosome accumulation, such as the one reported here, could have passed unnoticed in other organisms due to their transient nature. Future experiments will be required to fully address this issue and, in addition, to clarify the mechanisms underlying the localized formation of the extranucleolar body and the potential regulatory specificities of ribosome production at this site.

## MATERIALS AND METHODS

### Strains and plasmids

Strains used in this study are listed in Supplemental Table S1. Gene deletions and generation of alleles encoding green fluorescent protein (GFP)-tagged molecules were generated by one-step integration of PCR-amplified cassette sequences. The conditional strain carrying *CDC20* under the control of the MET3 promoter (YSG32) was constructed using the pE2017 integration plasmid (kind gift of Ethel Queralt, IDIBELL, Barcelona). Strains carrying the *CRM1* and *crm1(T539C)* (YGM198, YGM200), and *nup159-1* (YSG19) alleles were derived from strains (MNY7, MNY8, and LGY101) kindly provided by M. Rosbash (Brandeis University) and F. Estruch (University of Valencia), respectively.

Vectors utilized in this study are listed in Supplemental Table S2. Low-copy plasmids used to express untagged or GFP-tagged Arx1 (pGM63), Ltv1 (pGM64), Nop7 (pGM62), Pwp2 (pGM61), Rio2 (pGM65), and Rrp12 (pBN2, pGM37, pGM57, pLG1, and pLG2) under the NOP1 promoter were generated by cloning the corresponding PCR-amplified open reading sequences into the pRS316-NOP1-GFP backbone of pGFP-Ulp1 (kind gift of Vikram Panse, University of Zurich). Plasmids to conditionally express mCherry- and GFP-tagged Rrp12 variants (pGM4-5, pGM16, pGM34, and pGM51) under the GAL1 promoter were generated by cloning PCR-based cassettes into pGAL413 and pGAL415.

### Expression of fluorescent proteins and cell-cycle arrest conditions

Preparation of media, yeast transformation, and genetic manipulations were performed according to established procedures. Subcellular localization experiments of Tsr1-GFP in wild-type cells (Fig. 1A,D) were performed with the YGM1 strain. Results were further corroborated with a similar strain generated in the BY4743 genetic background. Subcellular localization experiments of Tsr1-GFP in cell-cycle progression defective cells (Fig. 1E–G) were performed on strains carrying the *MET-cdc20*, *esp1-1* or a *cdc*^*ts*^ mutant allele (YSG32, K2788, A5321, A2596, and Y850) and a plasmid for low-copy expression of Tsr1-GFP (pJB1). Subcellular localization experiments of *trans*-acting factors in wild-type cells (Fig. 2B) were performed with strains carrying integrated GFP-tagged alleles (YMD24, YMD6, YGM97, YGM1, YLG2, YLG1, YGM79, JDY851), a strain with the *RRP12* gene depleted that carried a low-copy plasmid for the expression of *GFP-rrp12* (YGM62), and W303 wild-type cells carrying low-copy plasmids for the expression of GFP-tagged factors (pNL25-T, pGM37, pGM61-65). Subcellular localization experiments of Net1-GFP (Fig. 3B,C) were performed in a wild-type strain carrying an integrated *GFP-net1* allele (YSG2) and a strain carrying both an *esp1-1* and an integrated *GFP-net1* allele (YSG13). The experiments of extranucleolar body formation in rDNA-tethering defective cells (Fig. 4A) were performed in strains carrying either a *heh1*Δ or a *csm1*Δ with an integrated *nop7-GFP* allele (YGM220, YGM218). The experiments of extranucleolar body formation in nucleoporin-defective cells (Fig. 4B) were performed in strains carrying a *nup*Δ or the *nup159-1* allele (Y05244, Y03551, Y00407, Y04917, and YGM192) and a low-copy plasmid for the expression of Nop7-GFP (pGM62).

For synchronization of wild-type strains in S phase, cells were treated with α-factor (7.5 mg/mL) for 3 h, washed, and cultured in media containing 200 mM hydroxyurea for 2 h. Cells were then washed and transferred to media with no drugs. Microscopy analyses were performed at 10-min intervals during 150 min upon release from the hydroxyurea arrest. For subcellular localization studies of Tsr1-GFP (driven from the pJB1 plasmid) in *esp1-1* and *cdc*^*ts*^ strains, cells were arrested with α-factor for 3 h at 24°C, washed, and released at 37°C for 150 min. In the case of the *MET-CDC20* strain, Tsr1-GFP localization (driven from the pJB1 plasmid) was visualized in cells transferred from methionine-free to methionine-supplemented media for 120 min. To monitor the formation of the extranucleolar body in *nup159-1* cells, we subjected cells carrying an integrated *nop7-GFP* allele (YSG19 strain) to the same synchronization-and-release scheme used for both the *esp1-1* and *cdc*^*ts*^ strains.

For the colocalization experiments of Rrp12 with either Tsr1 or Nop7 (Fig. 2C), YGM1 and JDY851 cells carrying the GAL1-driven pGM4-5 plasmid were transferred from 1.5% raffinose-0.5% glucose- to 2% galactose-containing media for 8 h before the α-factor-hydroxyurea synchronization procedure described above. The same strategy was used for the subcellular localization and proteomic studies of the Rrp12 deletion variants (Fig. 6C–E). To this end, we utilized wild-type (W303) cells carrying the GAL1-driven plasmids (pGM51, pGM16, and pGM34). For the colocalization experiments of Rrp12 with Spc42 (Fig. 2F) and Nop7 with Nup49 (Fig. 2E), strains SHM596-1 and JDY851 transformed with plasmids pGM37 and pUN100-Nup49-mCherry, respectively, were subjected to the α-factor-hydroxyurea arrest-and-release scheme described above.

For the colocalization studies involving the Nop7, Nup49, Net1, and Rrp12 proteins, we transformed *esp1-1* cells carrying integrated *nop7-GFP* (strain YGM15) or *net1-GFP* (strain YSG13) alleles with either the pUN100-Nup49-mCherry or the pGM4-5 plasmids. In the case of colocalization analyses of Rrp12 with Ulp1, we utilized *esp1-1* cells carrying both the pGM4-5 and the pUlp1-GFP plasmids. Cells were transferred from 1.5%-0.5% glucose- to 2% galactose-containing media for 8 h before being subjected to the α-factor synchronization at 24°C and release at 37°C as described above.

For Crm1 inactivation experiments, we used strains carrying either the wild-type *CRM1* or the *crm1-T539C* allele that expressed GFP-Rrp12 and Tsr1-GFP (driven from plasmids pJB1 and pGM57, respectively) or Nop7-GFP (expressed from an integrated allele in strains YGM198 and YGM200). Cells were arrested in S phase using the α-factor and hydroxyurea procedure described above and subsequently released in the presence or absence of 100 ng/mL leptomycin B (LMB). Two different time points were chosen for the treatments with leptomycin B, one just upon release from the arrest (referred to in Fig. 5A as LMB at 0 min), and the other one after 20 min of release from the arrest (referred to as LMB at 20 min)

### Standard fluorescence microscopy

For the detection of epifluorescence in vivo, cells were directly mounted onto microscope slides. DNA staining with 4′,6-diamidino-2-phenylindole was done in 4% formaldehyde-fixed cells as previously described (Dosil 2011) to score cells at different stages of the cell cycle. We considered that a protein was located in the extranucleolar body when its epifluorescence was detected accumulated in a distinctive punctate location in the daughter cell nucleus that was clearly separated from the nucleolus present in the mother cell side. The percentages of cells harboring the extranucleolar body were calculated after scoring 100 cells at the indicated time points or experimental conditions. To quantitate the fluorescence intensity, we measured the relative signal present in different nuclear regions and background zones in stacked microscopy cell projections using the Image J software (National Institutes of Health). To quantitate the Net1-GFP extranucleolar signal, we determined the total nuclear (T) and extranucleolar (EN) fluorescence intensities and, subsequently, obtained the ratio between the total (T) and the nucleolus (T minus EN) values. EN fluorescence was taken as the intranuclear fluorescence that was at least 0.2 µm distant from the bulk of the rDNA. For visualization of the mitotic spindle, we performed immunofluorescence studies with antibodies to tubulin as described elsewhere (Dosil 2011). Fluorescence microscopy was performed using either an Axioplan 2 (Zeiss) or an Olympus IX71 DeltaVision 6.2 (Applied Precision) microscope. For quantitation of the extranucleolar Net1-GFP (Fig. 3E,F), DAPI-stained fixed cells were imaged under low fluorescent excitation, to avoid rapid photobleaching, and 2× pixel binning to increase signal detection.

### Pre-rRNA fluorescence in situ hybridization (RNA FISH)

Cells were fixed in 4% formaldehyde at room temperature for 1 h and, after three washes in KS (100 mM K_2_HPO_4_ [pH 6.5], 1.2 M sorbitol) and a wash in KS-0.2% β-mercaptoethanol buffer, resuspended in KS-0.2% β-mercaptoethanol. Cells were then incubated with 25 µg/mL zymolyase 20T (Seikagaku), 0.02% glucuronidase (Sigma-Aldrich), 100 units/mL of RNasin (Promega), and 20 mM vanadyl ribonucleoside complex. After 20 min at 37°C, cells were washed three times in 100 mM potassium phosphate buffer (pH 6.5) containing 0.1% Igepal CO630, dehydrated in 70% ethanol for 30 min at −20°C, rehydrated in 2× SSC (0.30 M NaCl plus 0.030 M sodium citrate), and incubated overnight at 37°C with 1 µg/µL Cy3-conjugated oligonucleotide DA2 probe for the pre-rRNA D-A_2_ segment (5′-ATG CTC TTG CCA AAA CAA AAA AAT CCA TTT TCA AAA TTA TTA AAT TTC TT-3′; Sigma-Aldrich) in 2× SSC supplemented with 40% formamide, 4 mg/mL bovine serum albumin, RNasin (50 units/mL), 20 mM vanadyl ribonucleoside complex, 1 mg/mL salmon sperm DNA, and 1 mg/mL yeast tRNA. At the end of the hybridization, coverslips were washed twice in 2× SSC and 40% formamide for 10 min at 37°C, once in 2× SSC plus 0.1% Triton at 25°C, once in 2× SSC at 25°C, once in standard phosphate buffered saline solution and, finally, DNA was stained with 0.1 mg/mL 4′,6-diamidino-2-phenylindole. Samples were mounted in 90% glycerol containing p-phenylendiamine (1 mg/mL; Sigma-Aldrich) and analyzed under a microscopy as above. As controls for specificity of the extranucleolar signal observed in *esp1-1* cells, we performed parallel analyses with asynchronous wild-type cells, and Rrp12- or Rio2-depleted cells. As expected, the DA2-containing pre-rRNAs were mostly localized in the nucleolus in wild-type cells, and delocalized to the nucleoplasm and cytoplasm in Rrp12-depleted and Rio2-depleted cells, respectively. No discrete accumulation of those pre-rRNAs was observed outside the nucleolus.

### rDNA fluorescence in situ hybridization (DNA FISH)

Cell preparation and fluorescence in situ hybridization were performed exactly as described by Guacci et al. (1994) in a previous publication. The probe was prepared from a PCR-amplified DNA fragment encompassing nucleotides 38–4765 of the *RDN37-1* gene. This fragment contains the 5′ half of the rDNA repeat (5′-ETS1, 18S, ITS1, 5.8S, ITS2, and 5′-end of 25S). The probe was labeled with digoxigenin by nick translation using the DIG-Nick Translation Mix (Roche). The hybridized probe was detected by incubation with anti-digoxigenin monoclonal antibody (clone 1.71.256) (Roche).

### Complementation assays

The functional activity of Rrp12^198-1228^ and Rrp12^1070-1228^ was evaluated by examining cell growth and rRNA production in glucose-containing media of a GAL1-promoter conditional strain for *RRP12* (YPM7) transformed with low-copy plasmids for the expression of the corresponding Rrp12 fragments (pLG1, pLG2, and pBN2), as previously described (Moriggi et al. 2014).

### Proteomic analyses

Wild-type W303 cells carrying GAL1-driven plasmids (pGM51, pGM16, and pGM34) were transferred from raffinose- to galactose-containing media for 8 h, and Rrp12 proteins were immunoprecipitated using the GFP-Trap (Chromotek) technique. Subsequent electrophoretic separation, gel staining, and identification of Rrp12-binding proteins by mass spectrometry (Orbitrap, ThermoFisher) was performed using procedures described in detail in an earlier publication (Dosil 2011).

## SUPPLEMENTAL MATERIAL

Supplemental material is available for this article.

## Supplementary Material

Supplemental Material
