# Supplementary material for: Focal accumulation of preribosomes outside the nucleolus during metaphase–anaphase in budding yeast
Source: RNA. 2017 Sep;23(9):1432–43. doi: 10.1261/rna.061259.117 (PMC5558912; doi:10.1261/rna.061259.117)
Supplement: Supplemental Material [file supp_23_9_1432__index.html]

Focal accumulation of preribosomes outside the nucleolus during metaphase–anaphase in budding yeast — Supplemental Material 

# Focal accumulation of preribosomes outside the nucleolus during metaphase–anaphase in budding yeast

## Supplemental Material

- Supplemental\_Table\_S1.pdf
- Supplemental\_Table\_S2.pdf
