# Supplementary material for: Focal accumulation of preribosomes outside the nucleolus during metaphase–anaphase in budding yeast
Source: RNA. 2017 Sep;23(9):1432–43. doi: 10.1261/rna.061259.117 (PMC5558912; doi:10.1261/rna.061259.117)
Supplement: Supplemental Material [file supp_061259.117_Supplemental_Table_S1.pdf]

TABLE S1. Yeast strains used in this study

Name	Genotype*	Source
W303a	*MATa, ade2, his3, leu2, trp1, ura3*	Euroscarf
A2596	*MATa, ade2, his3, leu2, trp1, ura3, cdc15-2*	A. Amon (1)
A5321	*MATa, ade2, his3, leu2, trp1, ura3, cdc14-3*	A. Amon (1)
K2788	*MATa, ade2, leu2, ura3, esp1-1*	S. Elledge (2)
LGY101	*S288c, MATa, leu2, trp1, ura3, rat7-1*	C. Cole (3)
MNY7	*MATa, ade2, his3, leu2, trp1, ura3, crm1Δ::KANMX6 [pDC-CRM1 LEU2]*	M. Rosbash (4)
MNY8	*MATa, ade2, his3, leu2, trp1, ura3, crm1Δ::KANMX6 [pDC-crm1 (T539C) LEU2]*	M. Rosbash (4)
Y850	*MATa, ade2, his3, leu2, trp1, ura3, cdc5-1*	S. Elledge (2)
JDY851	*MATa, ade2, his3, leu2, trp1, ura3, nop7-GFP(S65T)::natNT2*	J. de la Cruz (5)
SHM596-1	*S288c, MATa, his3, leu2, trp1, ura3, spc42-EQFP611-KANMX6*	E. Schiebel (6)
Y00407	*BY4741, MATa, his3, leu2, met15, ura3, nup60Δ::KANMX4*	Euroscarf
Y03551	*BY4741, MATa, his3, leu2, met15, ura3, nup42Δ::KANMX4*	Euroscarf
Y04917	*BY4741, MATa, his3, leu2, met15, ura3, nup100Δ::KANMX4*	Euroscarf
Y05244	*BY4741, MATa, his3, leu2, met15, ura3, nup2Δ::KANMX4*	Euroscarf
YGM1	*MATa, ade2, his3, leu2, trp1, ura3, tsr1-GFP(S65T)::TRP1*	M. Dosil (7)
YGM15	*MATa, ade2, leu2, ura3, esp1-1, tsr1-GFP(S65T)-KANMX6*	This study
YGM62	*MATa, ade2, his3, leu2, trp1, ura3, rrp12Δ::KANMX4 [pGM37; pNOP1-GFP-rrp12 LEU2]*	This study
YGM79	*MATa, ade2, his3, leu2, trp1, ura3, nsa1-GFP(S65T)::natNT2*	This study
YGM97	*MATa, ade2, his3, leu2, trp1, ura3, enp1-GFP(S65T)::HIS3MX*	This study
YGM192	*MATa, his3, leu2, met15, ura3, nup116Δ::KANMX6*	This study
YGM198	*MATa, ade2, his3, leu2, trp1, ura3, nop7-GFP(S65T)::natNT2, crm1Δ::KANMX6 [pDC-CRM1 LEU2]*	This study

YGM200	*MATa, ade2, his3, leu2, trp1, ura3, nop7-GFP(S65T)::natNT2, crm1Δ::KANMX6 [pDC-crm1 (T539C) LEU2]*	This study
YGM218	*BY4741, leu2, met15, ura3, csm1Δ::KANMX6, nop7-GFP(S65T)::natNT2*	This study
YGM220	*BY4741, leu2, met15, ura3, heh1Δ::KANMX6, nop7-GFP(S65T)::natNT2*	This study
YLG1	*MATa, ade2, his3, leu2, trp1, ura3, rio2-GFP(S65T)::HIS3MX*	This study
YLG2	*MATa, ade2, his3, leu2, trp1, ura3, ltv1-GFP(S65T)::HIS3MX*	This study
YMD6	*MATa, ade2, his3, leu2, trp1, ura3, pwp2-GFP(S65T)::KANMX*	This study
YMD24	*MATa, ade2, his3, leu2, trp1, ura3, nop58-GFP(S65T)::TRP1*	This study
YPM7	*MATa, ade2, his3, leu2, trp1, ura3, KANM-GAL::HA-rrp12*	M. Dosil (7)
YSG2	*MATa, ade2, his3, leu2, trp1, ura3, net1-GFP(S65T)::natNT2*	This study
YSG13	*MATa, ade2, leu2, ura3, esp1-1, net1-GFP(S65T)-KANMX6*	This study
YSG19	*MATa, leu2, trp1, ura3, rat7-1(nup159-1), nop7-GFP(S65T)::natNT2*	This study
YSG32	*MATa, ade2, his3, leu2, trp1, ura3, CDC20::pMET3-3HA-cdc20-URA3, nop7-GFP(S65T)::natNT2*	This study

*Unless stated otherwise, strains are isogenic with strain W303

1. Tomson, B. N., D'Amours, D., Adamson, B. S., Aragon, L., and Amon, A. (2006) Ribosomal DNA transcription-dependent processes interfere with chromosome segregation. *Molecular and cellular biology* **26**, 6239-6247
2. Wang, Y., Shirogane, T., Liu, D., Harper, J. W., and Elledge, S. J. (2003) Exit from exit: resetting the cell cycle through Amn1 inhibition of G protein signaling. *Cell* **112**, 697-709
3. Gorsch, L. C., Dockendorff, T. C., and Cole, C. N. (1995) A conditional allele of the novel repeat-containing yeast nucleoporin RAT7/NUP159 causes both rapid cessation of mRNA export and reversible clustering of nuclear pore complexes. *The Journal of cell biology* **129**, 939-955
4. Neville, M., and Rosbash, M. (1999) The NES-Crm1p export pathway is not a major mRNA export route in Saccharomyces cerevisiae. *The EMBO journal* **18**, 3746-3756
5. Garcia-Gomez, J. J., Lebaron, S., Froment, C., Monsarrat, B., Henry, Y., and de la Cruz, J. (2011) Dynamics of the putative RNA helicase Spb4 during ribosome assembly in Saccharomyces cerevisiae. *Mol Cell Biol* **31**, 4156-4164
6. Sezen, B., Seedorf, M., and Schiebel, E. (2009) The SESA network links duplication of the yeast centrosome with the protein translation machinery. *Genes & development* **23**, 1559-1570
7. Moriggi, G., Nieto, B., and Dosil, M. (2014) Rrp12 and the Exportin Crm1 Participate in Late Assembly Events in the Nucleolus during 40S Ribosomal Subunit Biogenesis. *PLoS Genet* **10**, e1004836
