# Supplementary material for: Focal accumulation of preribosomes outside the nucleolus during metaphase–anaphase in budding yeast
Source: RNA. 2017 Sep;23(9):1432–43. doi: 10.1261/rna.061259.117 (PMC5558912; doi:10.1261/rna.061259.117)
Supplement: Supplemental Material [file supp_061259.117_Supplemental_Table_S2.pdf]

TABLE S2. **Plasmids used in this study**

**Name**	**Relevant information**	**Source**
pBN2	CEN, LEU2, pNOP1-rrp12$^{1070\text{-}1228}$	
pJB1	CEN, URA3, tsr1-GFP	S. Lemmon (1)
pNL25-T	CEN, TRP1, rpl25-GFP, mRFP-nop1	E. Hurt (2)
pGFP-Ulp1	CEN, LEU2, pNOP1-GFP-ulp1	V. Panse (3)
pGM4-5	CEN, HIS3, pGAL1-Rrp12-mCherry	This study
pGM16	CEN, LEU2, pGAL1-rrp12$^{1070\text{-}1228}$-GFP	This study
pGM34	CEN, LEU2, pGAL1-rrp12$^{198\text{-}1228}$-GFP	This study
pGM37	CEN, LEU2, pNOP1-GFP-rrp12	This study
pGM51	CEN, LEU2, pGAL1-rrp12-GFP	This study
pGM57	CEN, URA3, pNOP1-GFP-rrp12	This study
pGM61	CEN, LEU2, pNOP1-GFP-pwp2	This study
pGM62	CEN, LEU2, pNOP1-GFP-nop7	This study
pGM63	CEN, LEU2, pNOP1-GFP-arx1	This study
pGM64	CEN, LEU2, pNOP1-GFP-ltv1	This study
pGM65	CEN, LEU2, pNOP1-GFP-rio2	This study
pLG1	CEN, LEU2, pNOP1-rrp12	This study
pLG2	CEN, LEU2, pNOP1-rrp12$^{198\text{-}1228}$	This study
pUN100-Nup49-mCherry	CEN, LEU2, nup49-mCherry	V. Doye (4)

1. Gelperin, D., Horton, L., Beckman, J., Hensold, J. and Lemmon, S.K. (2001) Bms1p, a novel GTP-binding protein, and the related Tsr1p are required for distinct steps of 40S ribosome biogenesis in yeast. *RNA (New York, N.Y*, **7**, 1268-1283.
2. Ulbrich, C., Diepholz, M., Bassler, J., Kressler, D., Pertschy, B., Galani, K., Bottcher, B. and Hurt, E. (2009) Mechanochemical removal of ribosome biogenesis factors from nascent 60S ribosomal subunits. *Cell*, **138**, 911-922.
3. Panse, V.G., Kuster, B., Gerstberger, T. and Hurt, E. (2003) Unconventional tethering of Ulp1 to the transport channel of the nuclear pore complex by karyopherins. *Nat Cell Biol*, **5**, 21-27.
4. Chadrin, A., Hess, B., San Roman, M., Gatti, X., Lombard, B., Loew, D., Barral, Y., Palancade, B. and Doye, V. (2010) Pom33, a novel transmembrane nucleoporin required for proper nuclear pore complex distribution. *The Journal of cell biology*, **189**, 795-811.
